# Online Calibration of a Linear Micro Tomosynthesis Scanner

**DOI:** 10.3390/jimaging8100292

**Published:** 2022-10-21

**Authors:** Piroz Bahar, David Nguyen, Muyang Wang, Dumitru Mazilu, Eric E. Bennett, Han Wen

**Affiliations:** Laboratory of Imaging Physics, Biochemistry and Biophysics Center, National Heart, Lung and Blood Institute, National Institutes of Health, Bethesda, MD 20892, USA

**Keywords:** linear tomosynthesis, geometric calibration, histologic imaging, calcium scoring

## Abstract

In a linear tomosynthesis scanner designed for imaging histologic samples of several centimeters size at 10 µm resolution, the mechanical instability of the scanning stage (±10 µm) exceeded the resolution of the image system, making it necessary to determine the trajectory of the stage for each scan to avoid blurring and artifacts in the images that would arise from the errors in the geometric information used in 3D reconstruction. We present a method for online calibration by attaching a layer of randomly dispersed micro glass beads or calcium particles to the bottom of the sample stage. The method was based on a parametric representation of the rigid body motion of the sample stage-marker layer assembly. The marker layer was easy to produce and proven effective in the calibration procedure.

## 1. Introduction

### 1.1. Adaptation of Linear Tomosynthesis to Microscopy of Pathology Samples

**Figure 1:**
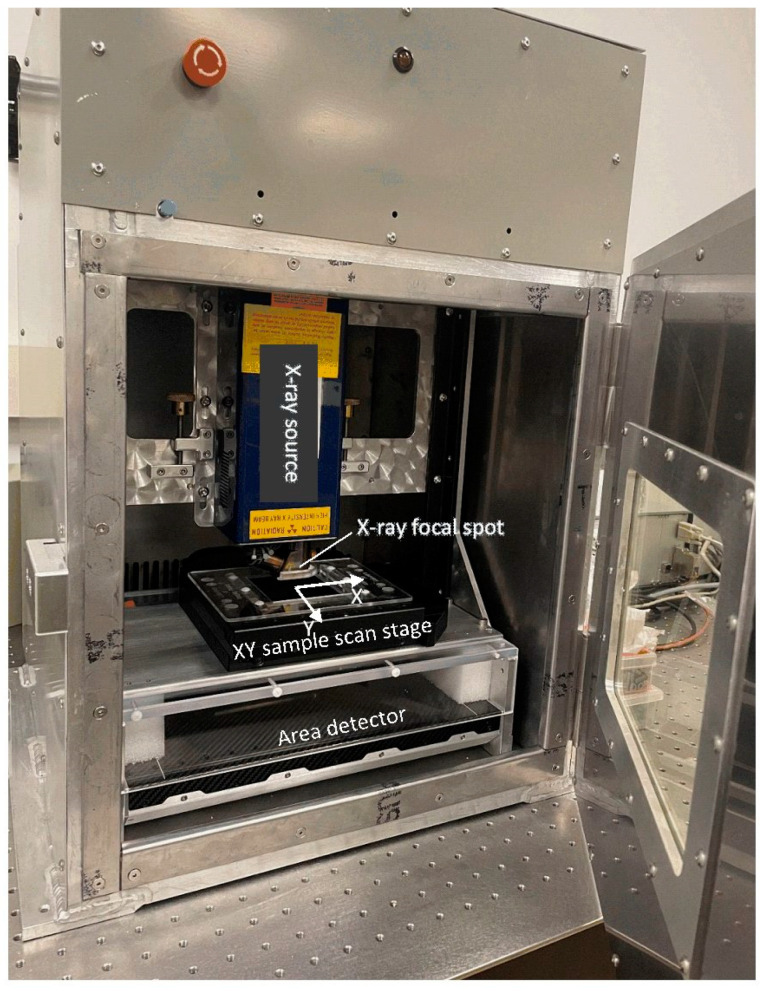
The X-ray micro tomosynthesis scanner with the radiation enclosure. The outer dimensions of the enclosure are 45 × 52 × 66 cm (width × depth × height). The motorized sample stage moves nominally in the horizontal plane. The sample is scanned horizontally across the vertical cone beam in either x or y direction. Flat samples are usually scanned in a horizontal plane near the X-ray focal spot to maximize the photon flux density through the sample and the geometric magnification for greater contrast-to-noise ration and resolution.

Tomosynthesis is an imaging technique that involves taking a series of X-ray images at different projection angles and reconstructing those images to form a stack of cross-sectional images at a range of depths [1]. It has the advantage of being able to resolve overlapping structures at different depths with relatively simple hardware. In the field of breast imaging, tomosynthesis has helped overcome challenges such as tissue superposition and false positive rates, thereby becoming widely accepted today [2,3]. Examples of other clinical applications besides mammography have also been published [4,5,6]. A particular type of linear tomosynthesis scan involves moving the sample down a straight line parallel to a flat panel detector [7,8,9,10]. It is related in concept to an early form of tomography called planigraphy [11]. In the field of non-destructive testing, it is sometimes called laminography, because it is particularly suited to scanning objects of flat shapes [7,8,10]. While this type of tomosynthesis was developed for materials testing and security screening, we were the first to realize that it was also advantageous for pathology samples that have flat shapes, such as standard paraffin embedding cassettes and tissue in Petri dishes. Therefore, we adapted it in the form of a linear micro tomosynthesis scanner for microscopy of tissue samples [12,13]. It provided 7 to 10 µm resolution with good soft-tissue contrast in a 15 min scan, which was an effective scouting tool to guide thin sectioning, staining and light microscopy [12,13]. The scanner is an X-ray cabinet system (Figure 1). The X-ray hardware is stationary, whereas the sample is carried on a motorized stage and scanned across a wide cone beam. The scanning geometry allows flat samples to be laid close to the X-ray source, to increase the geometric magnification and beam intensity through the sample. These factors then help to improve resolution and signal-to-noise ratio. Image reconstruction in tomosynthesis requires knowing the projection matrix from the 3D coordinates in the sample space to the 2D coordinates in the projection image. In practice, the geometry of the X-ray hardware and the sample deviate from the ideal design, and thus a calibration procedure is used to determine the deviations.

### 1.2. What Is New about the Present Calibration Method

Several recent methods have been published for the calibration of clinical tomosynthesis imaging systems [14,15,16,17,18,19]. These are based on offline scans of calibration phantoms. What is different about the current scanner is that the mechanical instability of the motorized sample stage (±10 µm) exceeded the resolution of the imaging system, and additionally the instability fluctuated with the scan range and speed. It meant that the geometric deviations were not repeatable, and needed to be calibrated online for each scan.

As a brief review of the literature, there is a wealth of calibration methods for cone-beam computed tomography (CT) and tomosynthesis imaging in diverse applications [14,15,16,17,18,19,20,21,22,23,24,25,26,27,28,29,30,31,32,33,34,35,36,37,38,39,40,41,42,43,44,45,46,47,48,49]. They can broadly be separated into offline phantom-based calibration methods and online phantom-less methods [39]. Graetz [47] and Jiang et al. [17] provided representative literature reviews of offline methods. For offline methods, the geometry of the imaging system is determined with dedicated scans of calibration phantoms. Calibration phantoms contain distinct positional markers, such as radio-opaquebeads [15,18,34,35,41,42,45,49] or wires [44]. Some phantom-based methods do not require knowledge of the positions of the markers in the phantom [23,25,35,36,45,47]. The second category of methods are online phantom-less calibration. These are based on the data from the sample scan itself, and do not require separate calibration scans or calibration phantoms [21,29,30,31,32,39,40]. To our knowledge, there were no published methods for online calibration of tomosynthesis scanners. Existing online calibration methods are phantom-less methods for CT scans of the full 180° or 360° projection angles. Unlike CT scans, tomosynthesis collects data from a limited range of projection angles and has inherent artifacts associated with the limited range. Therefore, it can be unreliable to measure geometric errors from artifacts or blurring of the sample itself, unless the sample contains an abundance of edges or points. Most of our pathology samples were soft tissue specimens that lack such sharp features.

From the above considerations, we created a hybrid method of online phantom-based calibration for tomosynthesis scans. The phantom is a dispersed layer of point makers (glass micro beads) placed at a sufficient distance below the sample platform, such that any tomosynthesis artifacts from the markers are confined in depth and do not interfere with the sample images. Our calibration algorithm has similarities to that of Stevens et al. for a circular-trajectory tomosynthesis scanner [23]. We operate on unfiltered back-projection images of the marker layer at and near focus. From the distribution of local deviations, we directly calculate the geometric parameters in a parametrized version of the sample stage movement. The parameters were then fed into the tomosynthesis reconstruction to improve the quality of the sample reconstruction.

The goal of the calibration is to reduce image blurring and preserve resolution. Since errors in the assumed movement of the sample stage lead directly to misalignment among the back-projected images in the image reconstruction process, which would lead directly to blurring when these back-projections were summed together to give the reconstructed image, calibration is verified based on the level of misalignment among all the back-projected images. The closer is the calibrated movement to the true movement, the less misalignment remains. The calibration is sufficient when the misalignment falls below the system resolution.

## 2. Materials and Methods

### 2.1. Scanner Hardware Geometry and Scan Procedure

Referring to Figure 1, the scanner consists of a stationary micro-focus X-ray tube and a stationary flat panel detector. In between the two, a motorized sample stage travels in a plane that is ideally parallel to the image plane of the detector, and in directions that are ideally parallel to the rows or columns of the pixel matrix of the detector. The geometric magnification factor of the system is generally between 10× and 16× depending on the sample thickness [13]. The X-ray tube had a focal spot of 5 μm at the operating condition of 30 kV/160 μA. The source-to-detector distance was 126 mm, and the source-to-sample distance was typically between 8 and 20 mm depending on the sample size. The flat panel X-ray detector had a pixel size of 74.8 μm and matrix size of 3072 × 3840 [13].

During a sample scan, the stage moves across the X-ray cone-beam at a constant speed in a straight line. The detector acquires projection images at a constant frame rate throughout the scan. As the sample traverses the cone beam, each point in the sample experiences X-rays of continually changing directions, which is equivalent to acquiring projection images from a range of angles. The maximum tomosynthesis angle is thus the angle subtended by the cone beam. A typical setting of scan range and speed for samples in standard tissue-embedding cassettes was 24.5 mm at 0.0272 mm/s, with a 15 min scan time.

Weighted filtered back-projection was used to reconstruct z-stacks of cross-sectional images at user specified ranges of depth [13]. A parametrized version of the sample stage movement was part of the input for the image reconstruction, which is described below.

### 2.2. Theoretical Basis of the Calibration Method

**Figure 2:**
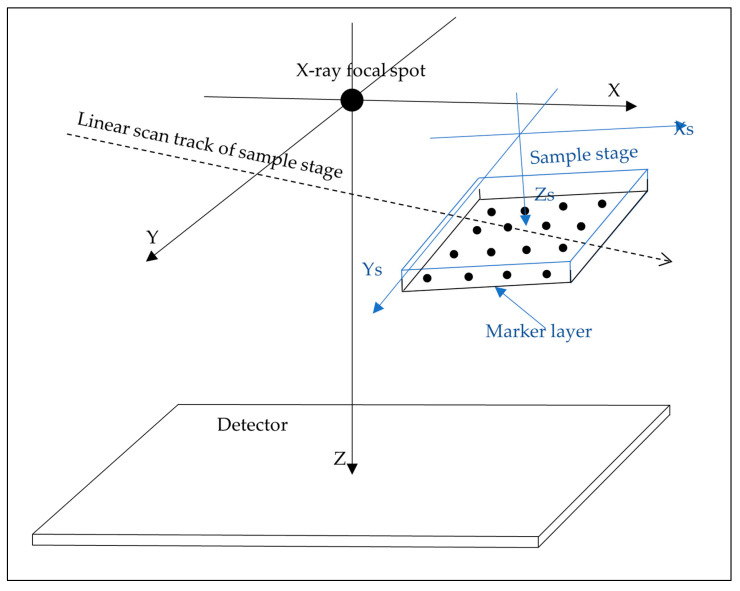
The definition of the sample coordinate system (X_s_, Y_s_, Z_s_) and the stationary scanner coordinate system (X, Y, Z). The sample coordinate system is attached rigidly to the sample stage and moves with it during the scan. The two coordinate systems coincide with each other at the mid time point of the scan. The glass microbead layer serving as fiducial markers for online geometric calibration is rigidly attached to the sample stage, typically at 1 cm below the sample. The scan track may not be perfectly aligned with the *X*-axis and the sample stage may rotate slightly during the scan. An exaggerated version of such deviations is illustrated.

The basic assumption is that the sample and the sample stage move as a rigid body during the scan. Referring to Figure 2, geometric calibration involves the transformation between two coordinate systems. The first is the stationary scanner coordinate system (x, y, z), with its origin at the X-ray focal spot, the *Z* axis pointing perpendicular to the detector, and the *X* and *Y* axes parallel to the rows and columns of the detector image matrix. The second is the sample coordinate system (x_s_, y_s_, z_s_), which travels with the sample stage. It is defined to coincide with the stationary scanner coordinate system at the midpoint of the scan. The ideal scan is a translational movement along the *X* axis. Generally, small deviations from the ideal movement have six degrees of freedom. These are functions of the travel distance *l* of the stage, including the 3D positional errors *d_x_*(*l*), *d_y_*(*l*) and *d_z_*(*l*), and small rotations of the sample stage represented by the three Euler angles *α*(*l*), *β*(*l*) and *γ*(*l*). By definition of the coordinate systems, the deviations are zero at the midpoint of the scan. We define *l* = 0 at the midpoint. To the leading order in the deviations, the general form of the transformation matrix from the sample to the scanner coordinates is

(1) $$\left(\begin{array}{c} x\\ y\\ z\\ 1 \end{array}\right)\approx \left(\begin{array}{cccc} 1 & -\gamma \left(l\right) & -\beta \left(l\right) & l+d_{x}\left(l\right)\\ \gamma \left(l\right) & 1 & -\alpha \left(l\right) & d_{y}\left(l\right)\\ \beta \left(l\right) & \alpha \left(l\right) & 1 & d_{z}\left(l\right)\\ 0 & 0 & 0 & 1 \end{array}\right)\left(\begin{array}{c} x_{s}\\ y_{s}\\ z_{s}\\ 1 \end{array}\right)$$

The deviations can be expanded in Taylor series of the travel distance *l*. Based on experimental data explained below, only the linear terms in *l* were significant relative to the resolution of our image system. Thus, the deviations are simplified to the form

(2) $$\left(\begin{array}{c} d_{x}\left(l\right)\\ d_{y}\left(l\right)\\ d_{z}\left(l\right)\\ \begin{array}{c} \alpha \left(l\right)\\ \beta \left(l\right)\\ \gamma \left(l\right) \end{array} \end{array}\right)\approx \left(\begin{array}{c} \delta_{x}\\ \delta_{y}\\ \delta_{z}\\ \begin{array}{c} \omega_{x}\\ \omega_{y}\\ \omega_{y} \end{array} \end{array}\right)l+O\left(l^{2}\right)$$

It is further simplified by dropping the term *δ_x_l* in the X direction, which is a scaling of the reconstructed images and does not affect image resolution. The *δ_y_* and *δ_z_* represent lateral deviations of the scan direction from the ideal X direction, and the *ω*’s represent the roll, pitch and yaw of the sample stage.

The projection matrix from the 3D sample coordinates to the 2D coordinates of the detector is given by

(3) $$\left(\begin{array}{c} x_{p}\\ y_{p} \end{array}\right)=\frac{SID}{z}\left(\begin{array}{c} x\\ y \end{array}\right)$$

where *SID* is the perpendicular distance from the focal spot to the image plane in the detector. Substituting Equation (2) into (1), and the results into (3), keeping only first order terms of the deviations and *l*, the 2D coordinates in the image plane are given by the expression

(4) $$\left(\begin{array}{c} x_{p}\\ y_{p} \end{array}\right)\approx \frac{SID}{z_{s}}\left(\begin{array}{c} x_{s}+l+O\left(\delta ,\omega \right)l\\ y_{s}+\delta_{y}l-z_{s}\omega_{x}l+x_{s}\omega_{z}l-y_{s}\frac{\delta_{z}}{z_{s}}l-x_{s}y_{s}\frac{\omega_{y}}{z_{s}}l-y_{s}^{2}\frac{\omega_{x}}{z_{s}}l \end{array}\right)$$

Equation (4) shows that for any fixed point in the sample, its projected 2D trajectory on the image plane may deviate from the ideal horizontal direction. The in-plane deviation angle varies with the 3D coordinates of the point in the sample coordinate system, which is given by the expression:

(5) $$\delta_{p}\left(x_{s},\;y_{s},z_{s}\right)\approx \delta_{y}-z_{s}\omega_{x}+x_{s}\omega_{z}-y_{s}\frac{\delta_{z}}{z_{s}}-x_{s}y_{s}\frac{\omega_{y}}{z_{s}}-y_{s}^{2}\frac{\omega_{x}}{z_{s}}$$

Equation (5) is the theoretical basis for the present calibration method. The next section explains how Equation (5) was observed experimentally for each sample scan, and how it was used to determine the parameters of the sample stage movement.

### 2.3. Fabrication of a Layer of Dispersed Markers

We used three protocols to create a dispersed layer of markers to be attached to the sample stage. One was dispersing glass microbeads of 20 µm diameter on the surface of a 1 mm thick acrylic plate by suspending both the microbeads and plate in water. The microbeads covered a square of 10 cm in size on the plate. The plate was rigidly attached to the sample stage at a level of 10 mm below the bottom surface. A second method was gluing a sheet of printer paper to the surface of an acrylic plate, whereby the cellulose fibers of the paper were the markers. The third method was dispersing a layer of hydroxyapatite powder on the acrylic plate and covering it with a 10 cm wide adhesive tape. Both the glass microbead and hydroxyapatite powder procedures produced a satisfactory marker layer, although the hydroxyapatite procedure was simpler. The printer paper had overly strong X-ray contrasts that made calculations unreliable.

### 2.4. Measurement of the Geometric Parameters

The overall geometric calibration procedure is a linear chain of 3 steps, consisting of: (A) measuring the in-plane tilt angle of the scan trajectories of the markers at different locations in the marker layer. This was carried out at a rectilinear grid of locations covering the entire field-of-view of the scan; (B) fitting the measurements of step A to the parametric model of Equation (5), from which the geometric parameters of the scan movement were extracted.; (C) applying the geometric parameters obtained in step B to image reconstruction of the sample. The following is a detailed description of the steps.

**Figure 3:**
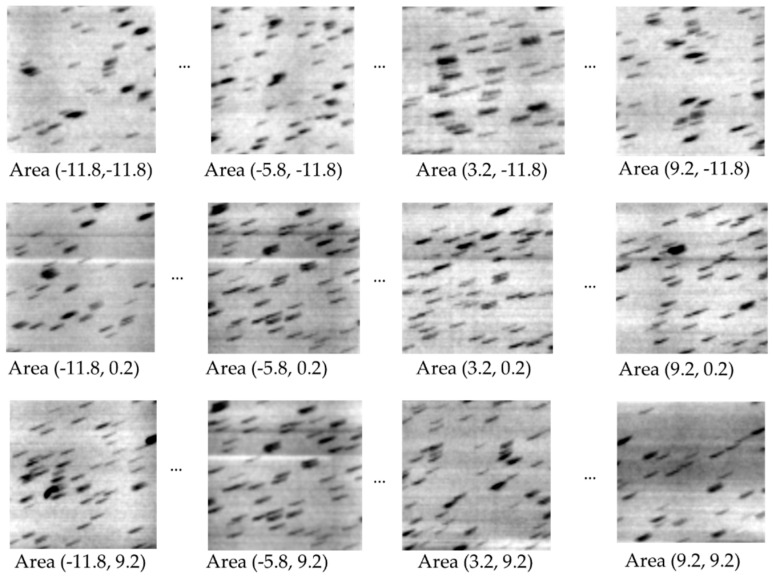
Illustration of how the in-plane tilt angle of the point markers varied with their locations in the field of view. The markers are glass microbeads. An array of reconstructed small areas of 1.4 × 1.4 mm size are shown, which are distributed on a rectangular grid across the full field-of-view. The numbers under each area are the x and y coordinates of the grid point in the sample coordinate system. The slices are off focus for the beads, such that each bead is defocused into a short line segment. The line segments are straight but tilted. The tilt is an effect of the deviations of the sample stage movement. The dependence of the tilt angle on the location of the beads provides sufficient information for estimating the 3D geometry of the scan.

To illustrate the quantity that is being measured in the first step, a z-stack of images centered at the depth of the marker layer was reconstructed assuming no geometric deviations. The z-stack covered a z range of 1 to 2 mm at 5 µm increment with an in-plane pixel size of 7 µm. In slightly off-focus images of the marker layer, the markers defocused into short line segments (Figure 3). The line segments were straight but tilted from the horizontal direction. The tilt represents an in-plane deviation of the projected trajectory of the bead from the *X* axis. The straightness of the line segments meant that the deviations of the scan movement were approximately linear with respect to the scan position. This was the basis for the linear approximation expressed by Equation (2). The in-plane deviation angle varied with the location of the beads, which is illustrated in Figure 3. This was anticipated by Equation (5).

**Figure 4:**
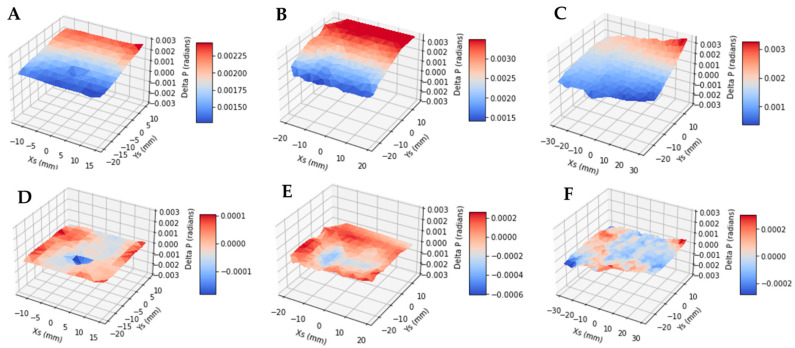
Surface plots of the measured in-plane deviation angles of the marker trajectories relative to the ideal horizontal direction, as a function of the marker position in the sample coordinate system. Plot (**A**) is from a scan of the typical speed of 0.0272 mm/s and range of 24 mm, (**B**) a scan of half the typical speed and range, and (**C**) a scan of twice the typical speed and range. Plots (**D**–**F**) are the residual deviation angles after subtracting the fit of the model of the scan geometry described by Equation (5) in Methods. The RMS of the residual deviation angles is <7.5% of the RMS of the original deviation angles. This indicates that the parametric model of Equation (5) adequately approximates the sample stage movement.

The first step of the calibration procedure was to obtain the in-plane deviation angle *δ_p_* of the marker trajectories as a function of the location of the markers. For this purpose, the entire marker layer was divided into a rectangular grid of 60 to 200 grid points. At each grid point, a small z-stack of 400 images covering a volume of 1.4 × 1.4 mm in plane and 2 mm depth was reconstructed around the z depth of the marker layer. The reconstruction was repeated while assuming a range of in-plane tilt angles of the scan trajectory, resulting in multiple small z-stacks. The in-plane deviation angle *δ_p_* at each grid point was determined as the scan trajectory angle that maximized the sharpness of the markers in the local z-stack. For each z-stack, marker sharpness was measured by the standard deviation of the image pixel values in the slice where the markers come into focus. That slice was determined as the one with the highest standard deviation value. At each grid point, among the multiple z-stacks of different scan trajectory angles, the scan trajectory angle giving the maximal marker sharpness was the sought-after deviation angle *δ_p_* for that grid point. This process was repeated for all grid points, and collectively provided the function *δ_p_*(x_s_, y_s_, z_s_) within the marker layer. Examples of the function is shown in the surface plots of Figure 4.

In the next step of the calibration, the measured function *δ_p_*(x_s_, y_s_, z_s_) from the marker layer was fitted to Equation (5) with polynomial fitting. The marker layer had a single depth z_s_, which reduced Equation (5) to a second-order function of the in-plane coordinates x_s_ and y_s_. The fitted coefficients of the 6 terms in Equation (5) then provided all the geometric parameters of the sample stage movement, including *δ_y_*, *δ_z_*, *ω_x_*, *ω_y_*, *ω_z_*. Lastly, these parameters were applied in the reconstruction of the sample images.

### 2.5. Assessing the Effect of Calibration by the Misalignment of Back-Projected Images

Since the goal of the calibration procedure is to reduce image blurring and improve image resolution, the effect of calibration is evaluated by the range of misalignment among the back-projected images, which is the direct cause of image blurring when the back-projected images are summed to produce the reconstructed image. Experimentally the misalignment is seen as linear drifts of the back-projected marker positions (Figure 3). The horizontal drift is equivalent to a slight change in the reconstructed depth of the markers and does not cause image blurring (see Equation (4)). The vertical drift causes image blurring. The range of the vertical drift, or vertical misalignment, is measured as

(6) $$M_{y}\left(x_{s},\;y_{s},z_{s}\right)=\frac{z_{s}}{SID}Disp_{y}\left(I\left(x_{p}\left(l=0\right),y_{p}\left(l=0\right)\right),\;I\left(x_{p}\left(l=L\right),y_{p}\left(l=L\right)\right)\right),$$

where in addition to the variables previously defined in Section 2.2, *L* is the total travel distance of the sample stage, *x_p_* and *y_p_* are the projected coordinates in the image plane based on the assumed movement of the sample stage through Equations (1)–(3), and *Disp_y_* is the y component of the displacement between the two images. This measurement was obtained for each small window in the field of view as defined in Section 2.4.

## 3. Results

As described in the Methods section, the key intermediate step of the calibration process is to determine the in-plane deviation angles of the marker trajectories from the prescribed scan direction, as a function of the location of the markers. Examples of the results for three different scan settings are shown Figure 4A–C. These figures are surface plots of the measured in-plane deviation angle as a function of the x_s_ and y_s_ coordinates of the markers. Variability of these plots illustrates the instability of the geometric parameters.

Table 1 summarizes the estimated geometric parameters for the three scan settings and a repeat scan of the first setting. The RMS of the residual of the deviation angles after subtraction of the model fit of Equation (5) was less than 7.5% of the measured values, indicating that the model approximates the actual distribution. The residual deviation angles of the marker trajectories as a function of the marker location are also plotted in Figure 4D,E.

**Figure 5:**
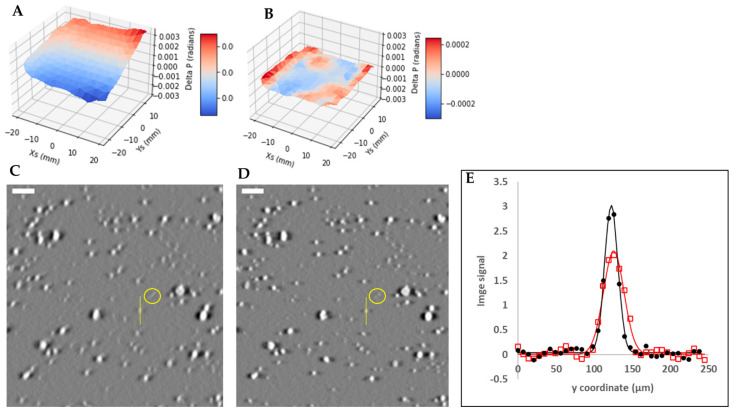
The effect of geometric calibration on the reconstructed image of a layer of hydroxyapatite particles. (**A**) is the surface plot of the deviation angles of the scan trajectories of the markers as a function of the marker position. The layer of the glass bead markers was fixed at 10 mm below the sample stage. (**B**) is the residual of the measurement in (**A**) after subtracting the fitted model of geometric calibration. (**C**,**D**) are the reconstructed images of the hydroxyapatite particles before and after applying the calibration. The yellow circles highlight the visible changes of the resolution of the particles. The scale bars are 160 µm. The yellow circles highlight two calcium specks that were only resolved after calibration. The line profiles across a calcium speck, marked by the yellow vertical lines in (**C**,**D**), are plotted in (**E**), where the red squares and red line are the measured signal profile and a Gaussian fit before calibration, and the black dots and black line are the corresponding quantities after calibration. The full width at half-maximum of the profiles were 31.7 and 20.5 µm before and after calibration, respectively.

**Figure 6:**
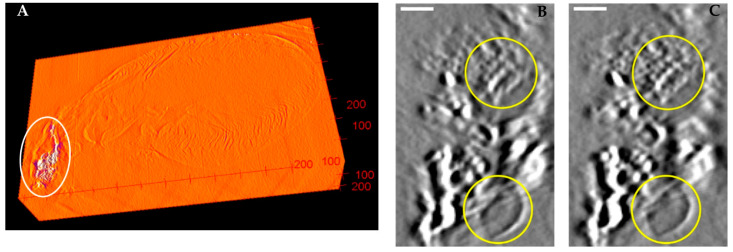
(**A**) is the reconstructed 3D stack of a paraffin-embedded mouse heart sample including an aorta segment. The white oval highlights a calcified lesion in the aorta. (**B**,**C**) are magnified views of the calcified lesion before and after applying geometric calibration. The white scale bar is equivalent to 160 µm. The yellow circles highlight the internal grain texture of the calcification and an air bubble.

Also summarized in Table 1 are the RMS of the range of misalignment of the marker positions among the back-projected images over the course of the scan, before and after applying the geometric parameters. The measurement is defined in Section 2.5 above. In all three scan settings, the range of misalignment was less than 7.2% of the un-calibrated levels. The post-calibration misalignment was below the system resolution of 7 µm in all settings. The effect of the calibration procedure on the reconstructed images is demonstrated in two samples. The first is a plexiglass plate coated with a dispersed layer of hydroxyapatite particles, and the second a paraffin-embedded mouse heart sample containing calcified atherosclerotic plaques in the aorta. The results are summarized in Figure 5 and Figure 6, respectively. In both cases, we observed an increase in the resolution of the reconstructed images after the calibration was applied.

## 4. Discussion

Since the tomosynthesis scanner described in this paper was designed to image tissue samples at 10 µm resolution, it was necessary to determine the precise movement and rotation of the sample stage each time a scan was performed, such that image reconstruction was corrected for minor mechanical instabilities. These instabilities were found to fluctuate from scan to scan (Table 1) and were significant enough to cause artifacts and blurriness in the resulting images if not corrected. Although the improvement can be subtle as in the example of Figure 6, the calibration procedure was needed to reach the true image resolution provided by the system hardware. 

Although the calibration method used a layer of point markers attached to the bottom of the sample stage, no particular pattern of the markers was required so far as they were dispersed throughout the imaging field of view. In practice, a layer of dispersed hydroxyapatite powder on a thin acrylic plate was relatively simple to make and effective for the procedure. A necessary condition is that the marker layer is rigidly attached to the sample stage, such that the two move as a rigid body. Another key point of the method is that there is adequate vertical separation between the sample and the marker layer to avoid mutual interference. For tissue samples in standard embedding cassettes, a 10 mm separation was found to be sufficient. The limitation of this method is the linear parametric model of the sample stage translation and rotation. It limits the utility of this method to situations where the scan movement is not significantly non-linear with respect to the distance of travel.

## Figures and Tables

**Table 1:** List of the calibrated geometric parameters for different scan settings. The RMS of the residual deviation angles of the scan trajectories of the markers after subtracting the model fit was <7.5% of the starting values. The residual range of misalignment of the marker positions in the back-projected images was based on reconstruction using the estimated geometric parameters. It was <7.2% of the uncalibrated levels.

	Typical Scan Speed and Range	Half Scan Speed and Range	Double Scan Speed and Range	Typical Scan Setting #2 Trial
*δ_y_* (radians)	1.76 × 10^−3^	3.98 × 10^−3^	1.58 × 10^−3^	1.92 × 10^−3^
*δ_z_* (radians)	−1.04 × 10^−3^	−1.25 × 10^−3^	−1.71 × 10^−3^	−1.66 × 10^−3^
*ω_x_* (radians/mm)	−6.88 × 10^−6^	3.18 × 10^−5^	−2.28 × 10^−6^	−5.17 × 10^−6^
*ω_y_* (radians/mm)	−1.18 × 10^−5^	−1.23 × 10^−5^	−4.14 × 10^−5^	−3.57 × 10^−5^
*ω_z_* (radians/mm)	−6.94 × 10^−7^	1.68 × 10^−6^	−2.30 × 10^−7^	−6.69 × 10^−7^
RMS of the deviation angles (radians)	1.83 × 10^−3^	2.80 × 10^−3^	1.66 × 10^−3^	1.98 × 10^−3^
RMS of the residual deviation angles after subtracting model fit (radians)	6.66 × 10^−5^	1.71 × 10^−4^	1.18 × 10^−4^	1.35 × 10^−4^
RMS of the range of misalignment of back-projected marker positions without calibration (µm)	45.75	35.01	83.18	50.04
RMS of the range of misalignment of back-projected marker positions with calibration (µm)	1.66	2.14	5.92	3.40

## Data Availability

The data presented in this study are available in this article.

## References

[1] Dobbins J.T., Godfrey D.J. (2003). Digital X-ray Tomosynthesis: Current State of the Art and Clinical Potential. Phys. Med. Biol..

[2] Vedantham S., Karellas A., Vijayaraghavan G.R., Kopans D.B. (2015). Digital Breast Tomosynthesis: State of the Art. Radiology.

[3] Chong A., Weinstein S.P., McDonald E.S., Conant E.F. (2019). Digital Breast Tomosynthesis: Concepts and Clinical Practice. Radiology.

[4] Gomi T., Nakajima M., Fujiwara H., Takeda T., Saito K., Umeda T., Sakaguchi K. (2012). Comparison between Chest Digital Tomosynthesis and CT as a Screening Method to Detect Artificial Pulmonary Nodules: A Phantom Study. Br. J. Radiol..

[5] Blum A., Noël A., Regent D., Villani N., Gillet R., Gondim Teixeira P. (2018). Tomosynthesis in Musculoskeletal Pathology. Diagn. Interv. Imaging.

[6] Machida H., Yuhara T., Tamura M., Ishikawa T., Tate E., Ueno E., Nye K., Sabol J.M. (2016). Whole-Body Clinical Applications of Digital Tomosynthesis. RadioGraphics.

[7] Zhou J., Maisl M., Reiter H., Arnold W. (1996). Computed Laminography for Materials Testing. Appl. Phys. Lett..

[8] Gondrom S., Zhou J., Maisl M., Reiter H., Kröning M., Arnold W. (1999). X-ray Computed Laminography: An Approach of Computed Tomography for Applications with Limited Access. Nucl. Eng. Des..

[9] Gao H., Zhang L., Chen Z., Xing Y., Xue H., Cheng J. (2013). Straight-Line-Trajectory-Based X-ray Tomographic Imaging for Security Inspections: System Design, Image Reconstruction and Preliminary Results. IEEE Trans. Nucl. Sci..

[10] O’Brien N.S., Boardman R.P., Sinclair I., Blumensath T. (2016). Recent Advances in X-ray Cone-Beam Computed Laminography. J. X-ray Sci. Technol..

[11] Howes W.E. (1939). Planigraphy—Its Application to Thoracic Diagnosis. Radiology.

[12] Wen H., Martinez A.M., Miao H.X., Larsen T.C., Nguyen C.P., Bennett E.E., Moorse K.P., Yu Z.X., Remaley A.T., Boehm M. (2018). Correlative Detection of Isolated Single and Multi-Cellular Calcifications in the Internal Elastic Lamina of Human Coronary Artery Samples. Sci. Rep..

[13] Nguyen D.T., Larsen T.C., Wang M., Knutsen R.H., Yang Z., Bennett E.E., Mazilu D., Yu Z.-X., Tao X., Donahue D.R. (2021). X-ray Microtomosynthesis of Unstained Pathology Tissue Samples. J. Microsc..

[14] Wang X., Mainprize J.G., Kempston M.P., Mawdsley G.E., Yaffe M.J. Digital Breast Tomosynthesis Geometry Calibration. Proceedings of the Medical Imaging 2007: Physics of Medical Imaging, SPIE.

[15] Li X., Zhang D., Liu B. (2010). A Generic Geometric Calibration Method for Tomographic Imaging Systems with Flat-Panel Detectors—A Detailed Implementation Guide. Med. Phys..

[16] Miao H., Wu X., Zhao H., Liu H. (2012). A Phantom-Based Calibration Method for Digital x-Ray Tomosynthesis. J. X-ray Sci. Technol..

[17] Jiang C., Zhang N., Gao J., Hu Z. (2017). Geometric Calibration of a Stationary Digital Breast Tomosynthesis System Based on Distributed Carbon Nanotube X-ray Source Arrays. PLoS ONE.

[18] Choi C.J., Vent T.L., Acciavatti R.J., Maidment A.D.A. Geometric Calibration for a Next-Generation Digital Breast Tomosynthesis System Using Virtual Line Segments. Proceedings of the Medical Imaging 2018: Physics of Medical Imaging, SPIE.

[19] Chang C.-H., Ni Y.-C., Huang S.-Y., Hsieh H.-H., Tseng S.-P., Tseng F.-P. (2019). A Geometric Calibration Method for the Digital Chest Tomosynthesis with Dual-Axis Scanning Geometry. PLoS ONE.

[20] Gullberg G.T., Tsui B.M.W., Crawford C.R., Ballard J.G., Hagius J.T. (1990). Estimation of Geometrical Parameters and Collimator Evaluation for Cone Beam Tomography. Med. Phys..

[21] Azevedo S.G., Schneberk D.J., Fitch J.P., Martz H.E. (1990). Calculation of the Rotational Centers in Computed Tomography Sinograms. IEEE Trans. Nucl. Sci..

[22] Noo F., Clackdoyle R., Mennessier C., White T.A., Roney T.J. (2000). Analytic Method Based on Identification of Ellipse Parameters for Scanner Calibration in Cone-Beam Tomography. Phys. Med. Biol..

[23] Stevens G.M., Saunders R., Pelc N.J. (2001). Alignment of a Volumetric Tomography System. Med. Phys..

[24] Beque D., Nuyts J., Bormans G., Suetens P., Dupont P. (2003). Characterization of Pinhole SPECT Acquisition Geometry. IEEE Trans. Med. Imaging.

[25] von Smekal L., Kachelriess M., Stepina E., Kalender W.A. (2004). Geometric Misalignment and Calibration in Cone-Beam Tomography. Med. Phys..

[26] Cho Y., Moseley D.J., Siewerdsen J.H., Jaffray D.A. (2005). Accurate Technique for Complete Geometric Calibration of Cone-Beam Computed Tomography Systems. Med. Phys..

[27] Yang K., Kwan A.L.C., Miller D.F., Boone J.M. (2006). A Geometric Calibration Method for Cone Beam CT Systems. Med. Phys..

[28] Hoppe S., Noo F., Dennerlein F., Lauritsch G., Hornegger J. (2007). Geometric Calibration of the Circle-plus-Arc Trajectory. Phys. Med. Biol..

[29] Panetta D., Belcari N., Guerra A.D., Moehrs S. (2008). An Optimization-Based Method for Geometrical Calibration in Cone-Beam CT without Dedicated Phantoms. Phys. Med. Biol..

[30] Kyriakou Y., Lapp R.M., Hillebrand L., Ertel D., Kalender W.A. (2008). Simultaneous Misalignment Correction for Approximate Circular Cone-Beam Computed Tomography. Phys. Med. Biol..

[31] Patel V., Chityala R.N., Hoffmann K.R., Ionita C.N., Bednarek D.R., Rudin S. (2009). Self-Calibration of a Cone-Beam Micro-CT System. Med. Phys..

[32] Kingston A., Sakellariou A., Varslot T., Myers G., Sheppard A. (2011). Reliable Automatic Alignment of Tomographic Projection Data by Passive Auto-Focus. Med. Phys..

[33] Li X., Zhang D., Liu B. (2011). Sensitivity Analysis of a Geometric Calibration Method Using Projection Matrices for Digital Tomosynthesis Systems. Med. Phys..

[34] Wu D., Li L., Zhang L., Xing Y., Chen Z., Xiao Y. Geometric Calibration of Cone-Beam CT with a Flat-Panel Detector. Proceedings of the 2011 IEEE Nuclear Science Symposium Conference Record.

[35] Sawall S., Knaup M., Kachelrieß M. (2012). A Robust Geometry Estimation Method for Spiral, Sequential and Circular Cone-Beam Micro-CT. Med. Phys..

[36] Gross D., Heil U., Schulze R., Schoemer E., Schwanecke U. (2012). Auto Calibration of a Cone-Beam-CT. Med. Phys..

[37] Wicklein J., Kunze H., Kalender W.A., Kyriakou Y. (2012). Image Features for Misalignment Correction in Medical Flat-Detector CT. Med. Phys..

[38] Ladikos A., Wein W. Geometric Calibration Using Bundle Adjustment for Cone-Beam Computed Tomography Devices. Proceedings of the Medical Imaging 2012: Physics of Medical Imaging, SPIE.

[39] Meng Y., Gong H., Yang X. (2013). Online Geometric Calibration of Cone-Beam Computed Tomography for Arbitrary Imaging Objects. IEEE Trans. Med. Imaging.

[40] Ben Tekaya I., Kaftandjian V., Buyens F., Sevestre S., Legoupil S. (2013). Registration-Based Geometric Calibration of Industrial X-ray Tomography System. IEEE Trans. Nucl. Sci..

[41] Xu M., Zhang C., Liu X., Li D. (2014). Direct Determination of Cone-Beam Geometric Parameters Using the Helical Phantom. Phys. Med. Biol..

[42] Zechner A., Stock M., Kellner D., Ziegler I., Keuschnigg P., Huber P., Mayer U., Sedlmayer F., Deutschmann H., Steininger P. (2016). Development and First Use of a Novel Cylindrical Ball Bearing Phantom for 9-DOF Geometric Calibrations of Flat Panel Imaging Devices Used in Image-Guided Ion Beam Therapy. Phys. Med. Biol..

[43] Zhou K., Huang Y., Meng X., Li Z., Li S., Yang K., Ren Q. (2016). A New Method for Cone-Beam Computed Tomography Geometric Parameters Estimation. J. Comput. Assist. Tomogr..

[44] Jacobson M.W., Ketcha M.D., Capostagno S., Martin A., Uneri A., Goerres J., Silva T.D., Reaungamornrat S., Han R., Manbachi A. (2018). A Line Fiducial Method for Geometric Calibration of Cone-Beam CT Systems with Diverse Scan Trajectories. Phys. Med. Biol..

[45] Li G., Luo S., You C., Getzin M., Zheng L., Wang G., Gu N. (2019). A Novel Calibration Method Incorporating Nonlinear Optimization and Ball-Bearing Markers for Cone-Beam CT with a Parameterized Trajectory. Med. Phys..

[46] Nguyen V., Sanctorum J.G., Van Wassenbergh S., Dirckx J.J.J., Sijbers J., De Beenhouwer J. (2021). Geometry Calibration of a Modular Stereo Cone-Beam X-ray CT System. J. Imaging.

[47] Graetz J. (2021). Auto-Calibration of Cone Beam Geometries from Arbitrary Rotating Markers Using a Vector Geometry Formulation of Projection Matrices. Phys. Med. Biol..

[48] Moon S., Choi S., Jang H., Shin M., Roh Y., Baek J. (2021). Geometry Calibration and Image Reconstruction for Carbon-Nanotube-Based Multisource and Multidetector CT. Phys. Med. Biol..

[49] Duan X., Cai J., Ling Q., Huang Y., Qi H., Chen Y., Zhou L., Xu Y. (2021). Knowledge-Based Self-Calibration Method of Calibration Phantom by and for Accurate Robot-Based CT Imaging Systems. Knowl.-Based Syst..

